# Loneliness, Social Integration, and Incident Dementia Over 6 Years: Prospective Findings From the English Longitudinal Study of Ageing

**DOI:** 10.1093/geronb/gbx087

**Published:** 2017-06-27

**Authors:** Snorri Bjorn Rafnsson, Martin Orrell, Eleonora d’Orsi, Eef Hogervorst, Andrew Steptoe

**Affiliations:** 1 Department of Epidemiology and Public Health, University College London, UK; 2 Institute of Mental Health, University of Nottingham, UK; 3 Department of Public Health, Universidade Federal de Santa Catarina, Florianópolis, Brazil; 4 School of Sport, Exercise and Health Sciences, Loughborough University, UK

**Keywords:** Dementia, Loneliness, Longitudinal, Social isolation, Social relationships

## Abstract

**Objectives:**

Social relationships are important for the maintenance of cognitive function at older ages, with both objective features of social networks and perceived social connections (loneliness) being relevant. There is limited evidence about how different aspects of social experience predict diagnosed dementia.

**Methods:**

The sample comprised 6,677 dementia-free individuals at baseline (2004) from the English Longitudinal Study of Ageing. Baseline information on loneliness, number of close relationships, marital status, and social isolation (contact with family and friends and participation in organizations) was analyzed in relation to incident dementia over an average 6.25 years using Cox regression, controlling for potential confounding factors.

**Results:**

Two hundred twenty participants developed dementia during follow-up. In multivariable analyses, dementia risk was positively related to greater loneliness (hazard ratio 1.40, 95% confidence interval 1.09–1.80, *p* = .008), and inversely associated with number of close relationships (*p* < .001) and being married (*p* = .018). Sensitivity analyses testing for reverse causality and different criteria for diagnosing dementia confirmed the robustness of these findings. There was no association with social isolation.

**Discussion:**

Dementia risk is associated with loneliness and having fewer close relationships in later life. The underlying mechanisms remain to be elucidated, but efforts to enhance older peoples’ relationship quality may be relevant to dementia risk.

Dementia is a global health problem and projections suggest the number of people with dementia worldwide may exceed 75 million by 2030 (Alzheimer’s Disease International, 2015). Although the incidence of Alzheimer’s disease and other forms of dementia appears to be declining (Satizabal et al., 2016), these problems make a major contribution to disability and health and social care needs among older people. Finding ways to prevent or delay dementia onset is a priority, and epidemiological and clinical evidence suggests that a broad range of biological and lifestyle factors influence risk (Norton, Matthews, Barnes, Yaffe, & Brayne, 2014). Social relationships are important in shaping health and wellbeing in later life, with greater social integration and larger networks of close relationships being associated with positive health outcomes (Holt-Lunstad, Smith, & Layton, 2010). Loneliness, which reflects the individual’s dissatisfaction with the frequency and closeness of relationships as distinct from objective indicators of social integration, is also relevant to functional decline and mortality risk (Holt-Lunstad, Smith, Baker, Harris, & Stephenson, 2015; Shankar, McMunn, Banks, & Steptoe, 2011).

There is substantial evidence that social integration is associated longitudinally with reduced rates of cognitive decline at older ages (Barnes, Mendes de Leon, Wilson, Bienias, & Evans, 2004; Bassuk, Glass, & Berkman, 1999; Ertel, Glymour, & Berkman, 2008), and social isolation may contribute to increased risk of dementia (Crooks, Lubben, Petitti, Little, & Chiu, 2008; Fratiglioni, Wang, Ericsson, Maytan, & Winblad, 2000; Saczynski et al., 2006; Stoykova, Matharan, Dartigues, & Amieva, 2011). By contrast, much of the literature relating loneliness with cognitive decline and dementia is cross-sectional (Boss, Kang, & Branson, 2015), and longitudinal evidence is limited (Shankar, Hamer, McMunn, & Steptoe, 2013; Tilvis et al., 2004; Wilson et al., 2007). Very few studies have investigated social integration and loneliness simultaneously (Holwerda et al., 2014). This is an important issue, since loneliness is distinct from objective social isolation and the two are only moderately correlated. There is evidence that they have different associations with health outcomes and mortality (Cornwell & Waite, 2009; Steptoe, Shankar, Demakakos, & Wardle, 2013). Greater understanding of the relevance of these different dimensions will aid population surveillance and the tracking of these processes over time. Furthermore, interventions designed to reduce isolation may have a different focus from those intended to alleviate loneliness and provide a greater sense of belonging (Cacioppo, Grippo, London, Goossens, & Cacioppo, 2015). In this study, we therefore tested whether loneliness and different aspects of social integration (marital status, number of close connections and a social isolation index) were associated both separately and in combination with dementia incidence over 6 years in a large population sample of older people in England. We tested whether these social relationship variables were associated with future dementia independently of baseline cognition, education, physical health, depression, mobility, and other risk factors.

Several pathways linking social relationships with dementia risk have been proposed (Fratiglioni, Paillard-Borg, & Winblad, 2004). First, stress-related processes could be involved. Socially isolated and lonely individuals may experience heightened exposure to stress in everyday life, and lack the social resources to buffer biological responses (Boss et al., 2015). Both isolation and loneliness have been associated with elevated cortisol in everyday life, and with heightened inflammatory cytokine responses to acute stress (Cacioppo, Cacioppo, Capitanio, & Cole, 2015; Grant, Hamer, & Steptoe, 2009; Hackett, Hamer, Endrighi, Brydon, & Steptoe, 2012). Disruption of hypothalamic-pituitary-adrenocortical activity may result in neural damage in frontal and limbic regions that in turn impairs cognitive function (McEwen, 2007), whereas systemic inflammation has been implicated in the neuropathological cascade culminating in dementia (Gorelick, 2010). Second, behavioral and lifestyle processes may be relevant. For example, social isolation and loneliness have been associated with the combination of reduced physical activity and smoking among older people, factors that contribute to cardiovascular disease risk (Shankar et al., 2011). Cardiovascular risk factors promote atherosclerotic small vessel disease and the neurofibrillary tangles characteristic of Alzheimer’s disease (Attems & Jellinger, 2014). A third possibility is that impoverished social relationships lead to a reduction in the number and quality of social interactions, and a diminution in cognitive stimulation, potentially leading to greater vulnerability to age-related neuropathological changes and cognitive decline (Bourassa, Memel, Woolverton, & Sbarra, 2017; Glei et al., 2005). These processes could affect cognitive reserve which in turn affects the association between beta-amyloid and cognitive decline (Yaffe et al., 2011). Recent brain imaging studies have documented an association between loneliness and high amyloid burden in cognitively unimpaired older men and women (Donovan et al., 2016).

There are major challenges in assessing the incidence of dementia in large-scale population studies in which detained clinical data are not available (Brayne & Davis, 2012). The primary analyses in the present study were based on physician diagnoses of dementia, together with impairments reported by informants for individuals who were not able to respond themselves. To address the issue that some cases of dementia will be missed, we carried out sensitivity analyses in which we augmented these criteria by identifying individuals who developed severely impaired cognitive function on objective tests over the study period.

## Methods

### Study Population

The English Longitudinal Study of Ageing (ELSA) is a population-based longitudinal panel study of a representative sample of initially noninstitutionalized men and women aged 50 and older living in England, designed to explore a range of social, economic, biological and psychological factors relevant to aging. It began in 2002 (Wave 1), with repeat assessments every 2 years (Steptoe, Breeze, Banks, & Nazroo, 2013). The baseline for the present analysis was Wave 2 (2004) since that was the wave in which a measure of loneliness was first introduced. Outcomes were assessed in Waves 3 (2006), 4 (2008), 5 (2010), and 6 (2012). The primary form of data collection in ELSA is a computer assisted personal interview (CAPI) carried out face to face in the person’s home or residence. Additional data are obtained from self-completion questionnaires that respondents return to the research office by mail after the CAPI, typically with an 88%–90% response rate. In Wave 2, 8,780 participants took part in the face-to-face interview; 101 were excluded from subsequent analyses as already having dementia; 7,680 participants returned the self-completion questionnaire with questions about loneliness, social isolation, and number of close relationships. Two hundred thirty-three deaths occurred between Wave 2 and Wave 3, 104 participants had missing data on covariates, and 656 had no data on dementia at any point during follow-up. Individuals not in the analyses were relatively older, less educated, less affluent, and had poorer cognition and fewer close relationships than those included in the study. The analytic sample of 6,677 consisted of 2,961 men and 3,716 women, with a mean age of 66.0 ± 9.4 (*SD*) ranging from 52 to >90 years at baseline. Ethical approval was granted from the National Research and Ethics Committee (http://www.nres.npsa.nhs.uk/), and all participants provided written consent.

### Measures

#### Dementia assessment

Dementia was defined as a physician diagnosis of dementia or Alzheimer’s disease reported by the participant during the CAPI. When an individual was not able to participate personally because of incapacity, a family member or long-term carer completed an adapted short-form IQCODE questionnaire (Jorm, 1994). This consists of 16 items asking the informant to comment on the ability of the person compared with 2 years ago to perform various functions (e.g., remembering the names of family members) with ratings ranging from *much improved* to *much worse*. We used the cut-off point of 3.5 to define dementia as this has high specificity and good sensitivity (Quinn et al., 2014). The primary analyses were based on the combination of a positive physician diagnosis or an IQCODE rating above threshold.

#### Augmented dementia assessment

There were two cognitive tests that were included in all waves of ELSA: memory (immediate and delayed recall), and time orientation. In the memory assessment, participants were presented with a list of 10 words that were read out at the rate of one word every 2 seconds. A total of four such lists were available, and these were randomly allocated. Participants recalled as many words as they could both immediately and after an interval during which they completed other cognitive function tests (delayed recall). The two scores were combined. Time orientation was assessed using four questions relating to day and date from the Mini-Mental State Examination (Folstein, Folstein, & McHugh, 1975). We established that a score of 2, 1, or 0 on either test was >2 *SD*s below the population mean. Individuals with scores ≤2 in both domains were defined as possible dementia cases.

#### Loneliness and social relationships

Social isolation was assessed with an index including extent of contact with the person’s social network and involvement in social organizations (Shankar et al., 2011; Steptoe, Shankar, Demakakos, & Wardle, 2013). Participants were asked about the extent of contact with three categories of social tie: children, family apart from spouse and children (e.g., cousins), and friends. The following categories were used in response: less than once a year or never, once or twice a year, every few months, once or twice a month, once or twice a week, and three or more times a week. Based on the thresholds described by Cohen et al. (1997), we gave a point if the respondent had less than monthly contact (including face-to-face, telephone or written/e-mail contact) with each category or social tie. Participants were given an additional point if they did not participate in any organizations such as social clubs, sports clubs, churches or residents’ groups. Scores ranged from 0 to 4, with higher scores indicating greater social isolation. The number of participants scoring 4 was small, so categories 3 and 4 were combined. Marital status was excluded from this index but entered into analyses as a separate variable, since there is already an established literature relating marital status with dementia (Fratiglioni et al., 2000; Hakansson et al., 2009).

We measured loneliness with the three-item, short form of the Revised UCLA loneliness scale (Hughes, Waite, Hawkley, & Cacioppo, 2004). Ratings were averaged to produce a loneliness score ranging from 1 to 3. Number of close relationships was computed by asking respondents the number of children, other family, and friends with whom they have a close relationship. Responses were summed and grouped into five categories (0–1, 2–3, 4–5, 6–9, and 10 or more).

#### Covariates

We indexed socioeconomic status by total household wealth net of debt. Wealth is a robust indicator of socioeconomic circumstances and standard of living in ELSA (Steptoe, Breeze, Banks, & Nazroo, 2013), and was divided into deciles for the purposes of analysis. Educational attainment was divided into three categories: no formal qualifications, intermediate (equivalent to junior high school and high school) and higher education (college education). Marital status was classified into married or equivalent versus other (never married, divorced, separated, or widowed). Physician diagnoses of coronary heart disease (CHD), cancer, stroke, diabetes, and hypertension were also collected, since these may be relevant to future dementia risk. Mobility was defined by asking respondents whether they had difficulties with one or more 10 common leg and arm functions (e.g., walking 100 yards). Baseline cognition was assessed by amalgamating scores from four cognitive tests assessing memory (immediate and delayed recall of word list), semantic verbal fluency (animal naming over 1 min), and attention and processing speed (speed and accuracy on a letter cancellation task). We computed normalized *z* scores for each test and averaged the normalized scores across tests to produce a single measure. We assessed depressive symptoms using the 8-item Centre for Epidemiologic Studies Depression Scale (CES-D). The item on loneliness was omitted from the CES-D to avoid direct overlap with the loneliness measure, and a score of 6 or more was used to define severe depressive symptoms.

### Statistical Analysis

We used Cox proportional hazards regression models to estimate hazard ratios (HR) of dementia incidence and 95% confidence intervals (CIs), with survival time being measured in months from date of the Wave 2 interview to onset of dementia or to follow-up in Wave 6 (2012/2013). For individuals who died (*n* = 856) or dropped out of the study without dementia, the latest wave of data collection was used as the census point. We fitted five models. Model 1 included all covariates measured at baseline plus marital status. We added social isolation in Model 2, loneliness in Model 3 and number of close relationships in Model 4. Model 5 included all the social relationship variables along with the covariates. We performed collinearity diagnostic tests to check that collinearity was not present, and variable inflation factors were <1.5.

We carried out five sensitivity analyses. The first addressed the issue of reverse causality by excluding cases diagnosed 24 months or 48 months from the baseline assessment, in case marked decline in the months before a diagnosis led to social withdrawal or changes in patterns of close relationships. The second sensitivity analysis used binary logistic regression instead of Cox modeling, since the date of diagnosis was often not precise. In the third sensitivity analysis, we excluded participants who had died from Wave 3 onwards, in case proximity to death during the study period modified associations. The fourth sensitivity analysis involved the augmented definition of dementia, including very low cognitive performance along with physician diagnoses and IQCODE scores above threshold. The fifth set of sensitivity analyses related to the computation of the social isolation index. First, marital/cohabiting status was added to the index, instead of modeling it as a separate variable. Then we recomputed the index after changing the threshold of frequency of contact with children, other family, and friends. In separate analyses, the threshold of contact was changed from at least monthly contact to at least weekly or less than weekly (a higher intensity level of social interaction), and to more or less than every few months (a lower intensity level of social interaction). All statistical tests were two-tailed, with *p* < .05 taken as significant. Analyses were performed using IBM SPSS Statistics V 22 and Stata SE13.

## Results

Of the 6,677 participants free of dementia at baseline, 220 (3.3%) were diagnosed with dementia (*n* = 172) or had an informant rating above threshold (*n* = 48) during the average 6 year, 3-month follow-up period. The dementia group included 88 men (3.0%) and 132 women (3.5%). At baseline, participants who developed dementia were older on average, had less education, and less wealth than those who remained without dementia (Table 1). The proportion of individuals who developed dementia in each age band was 0.6% (52–59 years), 1.3% (60–69 years), 5.4% (70–79 years), and 13.9% (≥80 years), with an age-adjusted incidence over the 6.25-year period of 3.3% overall, and 5.8% in participants aged ≥65 years. Dementia cases were also more likely to have hypertension, diabetes, stroke, and CHD (all *p* < .001). The dementia group had relatively poorer cognitive function at baseline (*p* < .001) and were more likely to have impaired mobility (*p* < .001). The univariate analyses indicated that individuals in the future dementia group were less likely to be married (*p* = .018), had fewer close relationships (*p* < .001), and reported greater loneliness (*p* < .001) but that there were no differences in the social isolation index. The associations between the social relationship variables are detailed in Supplementary Table 1. Measures were only moderately correlated, and the strongest association was between loneliness and being married (*r* = −.31).

**Table 1.** Baseline Characteristics of Participants With and Without Dementia on Follow-up

	No dementia (*n* = 6,457)	Dementia (*n* = 220)	*p* difference
Sex: Men	2,873 (44.5%)	88 (40.0%)	.19
Women	3,584 (55.5%)	132 (60.0%)	
Age: 52–59 years	2,091 (32.4%)	13 (5.9%)	.001
60–69	2,288 (35.4%)	31 (14.1%)	
70–79	1,529 (23.7%)	87 (39.5%)	
≥80	549 (8.5%)	89 (40.5%)	
Education: Lower	2,278 (35.3%)	119 (54.1%)	.001
Intermediate	2,473 (38.3%)	67 (30.5%)	
Higher	1,706 (26.4%)	34 (15.5%)	
Wealth (decile)	5.90 ± 2.8	5.10 ± 2.9	.001
Hypertension	2,776 (43.0%)	130 (59.1%)	.001
Diabetes	518 (8.0%)	35 (15.9%)	.001
Stroke	242 (3.7%)	28 (12.7%)	.001
Coronary heart disease	718 (11.1%)	45 (20.5%)	.001
Cancer	468 (7.2%)	21 (9.5%)	.19
Impaired mobility	3,682 (57.0%)	169 (76.8%)	.001
Depression	227 (4.4%)	8 (4.3%)	.98
Cognition index	0.07 ± 0.61	−0.54 ± 0.65	.001
Marital status: Married	4,409 (68.3%)	133 (60.5%)	.018
Not married	2,048 (31.7%)	87 (39.5%)	
Social isolation: 0	3,899 (60.4%)	130 (59.1%)	.73
1	2,057 (31.9%)	74 (33.6%)	
2	423 (6.6%)	12 (5.5%)	
3	78 (1.2%)	4 (1.8%)	
Loneliness	1.37 ± 0.50	1.54 ± 0.57	.001
Close relationships: 0–1	287 (4.4%)	30 (13.6%)	.001
2–3	720 (11.2%)	37 (16.8%)	
4–5	1,189 (18.4%)	38 (17.3%)	
6–9	2,481 (38.4%)	63 (28.6%)	
≥10	1,780 (27.6%)	52 (23.6%)	

*Note*: *N* (%) and Means ± *SD*.

### Associations Between Social Relationship Variables and Incident Dementia

Model 1 of the Cox regression showed that marital status was associated with dementia, with a HR of 1.77 (95% CI 1.29–2.44) for unmarried compared with married participants (Table 2). Social isolation was not an independent predictor of dementia incidence in Model 2. However, greater loneliness was associated with future dementia risk (Model 3, adjusted HR 1.44, 95% CI 1.11–1.88, *p* = .006). There was a 44% increase in the risk of future dementia for every unit change in loneliness rating independent of covariates. Model 4 indicated that, compared with individuals who reported no or only one close relationship, the risk of dementia was 0.43 in those reporting 2–3 close relationships, 0.38 for people with 4–5 close relationships, 0.34 for people with 6–9, and 0.32 for 10 or more close relationships (all *p* < .001). Model 5 introduced all the social relationship variables simultaneously, along with covariates. Marriage, loneliness, and number of close relationships remained independent predictors of dementia, with a small reduction in the strength of the association for loneliness (hazards ratio change from 1.44 to 1.33). Other factors independently predicting dementia in the final combined model were baseline older age, hypertension, low cognitive ability, and not being married.

**Table 2.** Cox Proportional Hazards Regressions of the Incidence of Dementia (2006–2012) on Social Relationship Measures

	Model 1	Model 2	Model 3	Model 4	Model 5
Sex^a^	1.22 (0.90 to 1.64)	1.22 (0.91–1.65)	1.20 (0.89–1.61)	1.25 (0.93–1.69)	1.23 (0.92–1.66)
Age: 52–59 years	1 [ref]	1 [ref]	1 [ref]	1 [ref]	1 [ref]
60–69	1.74 (0.91 to 3.34)	1.74 (0.91–3.34)	1.77 (0.92–3.41)	1.72 (0.90–3.30)	1.75 (0.91–3.37)
70–79	6.20 (3.41 to 11.24)*	6.25 (3.44–11.36)*	6.39 (3.52–11.62)*	6.05 (3.33–11.00)*	6.21 (3.41–11.28)*
≥80	18.31 (9.87 to 33.96)*	18.56 (9.96–34.37)*	18.56 (10.06–34.62)*	17.91 (9.66–33.21)*	18.37 (9.89–34.13)*
Education: Lower	1 [ref]	1 [ref]	1 [ref]	1 [ref]	1 [ref]
Intermediate	0.91 (0.67–1.25)	0.92 (0.67–1.26)	0.92 (0.67–1.26)	0.91 (0.67–1.25)	0.93 (0.68–1.27)
Higher	0.90 (0.59–1.37)	0.92 (0.60–1.40)	0.89 (0.58–1.36)	0.91 (0.60–1.39)	0.92 (0.60–1.41)
Wealth (decile)	0.97 (0.92–1.03)	0.97 (0.92–1.03)	0.98 (0.92–1.03)	0.98 (0.93–1.03)	0.98 (0.93–1.04)
Hypertension^b^	1.31 (0.99–1.73)	1.30 (0.98–1.71)	1.32 (0.99–1.74)	1.34 (1.02–1.77)*	1.34 (1.01–1.77)*
Diabetes^b^	1.29 (0.88–1.88)	1.30 (0.89–1.90)	1.34 (0.92–1.97)	1.27 (0.87–1.86)	1.32 (0.90–1.92)
Stroke^b^	1.29 (0.84–1.97)	1.29 (0.84–1.97)	1.29 (0.84–1.98)	1.29 (0.85–1.98)	1.30 (0.85–1.99)
Coronary heart disease^b^	1.26 (0.90–1.77)	1.25 (0.89–1.77)	1.22 (0.86–1.72)	1.27 (0.90–1.79)	1.24 (0.88–1.75)
Cancer^b^	1.06 (0.67–1.68)	1.06 (0.67–1.68)	1.08 (0.68–1.70)	1.08 (0.68–1.71)	1.10 (0.69–1.74)
Mobility^c^	1.20 (0.86–1.68)	1.20 (0.86–1.69)	1.14 (0.81–1.60)	1.23 (0.88–1.72)	1.17 (0.84–1.65)
Depression^d^	1.02 (0.54–1.95)	0.97 (0.50–1.86)	0.79 (0.41–1.55)	1.04 (0.54–1.98)	0.82 (0.41–1.62)
Cognition	0.32 (0.26–0.39)*	0.32 (0.26–0.39)*	0.33 (0.27–0.41)*	0.33 (0.26–0.40)*	0.33 (0.27–0.41)*
Marital status^e^	1.77 (1.29–2.44)*	1.77 (1.29–2.44)*	1.96 (1.41–2.71)*	1.98 (1.43–2.73)*	2.11 (1.52–2.92)*
Social isolation: 0		1 [ref]			1 [ref]
1		1.19 (0.89–1.58)			1.11 (0.83–1.48)
2		0.96 (0.52–1.76)			0.88 (0.48–1.62)
3		1.50 (0.54–4.24)			1.22 (0.44–3.40)
Loneliness			1.44 (1.11–1.88)*		1.33 (1.02–1.73)*
Close relationships: 0–1				1 [ref]	1 [ref]
2–3				0.43 (0.26–0.70)*	0.43 (0.26–0.70)*
4–5				0.38 (0.23–0.61)*	0.39 (0.24–0.64)*
6–9				0.34 (0.22–0.53)*	0.36 (0.23–0.57)*
≥10				0.32 (0.20–0.51)*	0.35 (0.22–0.56)*

*Notes*: Adjusted hazards ratios with 95% confidence intervals. *N* = 6,677.

^a^Male is the reference group.

^b^No illness is the reference group.

^c^No mobility impairment is the reference group.

^d^Low depressive symptoms is the reference group.

^e^Married is the reference group.

*p < .05.

### Sensitivity Analyses

The first sensitivity analyses excluded cases of dementia diagnosed within 24 months of baseline assessments, then cases diagnosed 48 months or less from baseline. The number of dementia cases fell from 220 to 185 in the first step, whereas the second step left only 127 cases, further reducing the statistical power in the models. Nevertheless, in both instances, loneliness and number of close relationships remained independently associated with dementia onset over the follow-up period (Table 3). Thus, it appears that the association between social relationship variables and dementia did not depend on the development of dementia within a relatively short time after baseline assessments. The second sensitivity analysis replicated the findings of the proportional hazards regressions with binary logistic regression. Model 5 is detailed in Supplementary Table 2, where it is evident that marriage (odds ratio [OR] 1.92. 95% CI 1.36–2.71), loneliness (OR 1.36, 95% CI 1.03–1.80) and number of close relationships (OR 0.52 to 0.39) were independently associated with dementia risk after adjustment for all covariates. The third sensitivity analysis excluded people who died during the study period, leaving a study sample of 5,526 with 142 dementia cases. As shown in Supplementary Table 3, the key associations between dementia incidence and loneliness and number of close relationships were maintained.

**Table 3.** Cox Proportional Hazards Regressions of the Incidence of Dementia (2006–2012) on Social Relationship Variables, Excluding Cases in the 24 and 48 Months After Baseline

	Excluding cases in the 24 months after baseline *N* = 5,352		Excluding cases in the 48 months after baseline *N* = 4,778	
	Adjusted hazards ratio (95% CI)	*p*	Adjusted hazards ratio (95% CI)	*p*
Sex^a^	1.33 (0.96–1.85)	.089	1.58 (1.05–2.36)	.027
Age: 52–59 years	1		1	
60–69	1.87 (0.92–3.79)	.083	2.41 (0.96–6.08)	.062
70–79	6.72 (3.52–12.86)	<.001	9.37 (3.96–22.18)	<.001
≥80	20.58 (10.46–40.31)	<.001	33.17 (13.6–80.93)	<.001
Education: Lower	1		1	
Intermediate	0.98 (0.70–1.38)	.98	1.12 (0.75–1.68)	.59
Higher	0.99 (0.63–1.57)	.99	0.94 (0.53–1.67)	.83
Wealth (decile)	0.98 (0.92–1.04)	.56	0.99 (0.92–1.06)	.76
Hypertension^b^	1.30 (0.96–1.76)	.093	1.55 (1.07–2.25)	.020
Diabetes^b^	1.42 (0.94–2.15)	.099	1.37 (0.82–2.28)	.23
Stroke^b^	1.07 (0.65–1.77)	.79	0.94 (0.49–1.79)	.84
Coronary heart disease^b^	1.33 (0.91–1.94)	.14	1.16 (0.71–1.90)	.54
Cancer^b^	0.95 (0.56–1.61)	.86	1.18 (0.66–2.09)	.58
Mobility^c^	1.10 (0.76–1.58)	.63	1.04 (0.67–1.61)	.87
Depresssion^d^	0.76 (0.35–1.64)	.49	0.59 (0.20–1.71)	.33
Cognition	0.34 (0.27–0.42)	<.001	0.32 (0.24–0.43)	<.001
Marital status^e^	1.97 (1.38–2.82)	<.001	2.29 (1.47–3.56)	<.001
Social isolation: 0	1		1	
1	1.17 (0.85–1.61)	.32	0.92 (0.12–6.87)	.33
2	1.01 (0.53–1.92)	.97	1.39 (0.67–2.88)	.93
3	1.24 (0.38–4.04)	.78	1.39 (0.95–2.03)	.38
Loneliness	1.45 (1.09–1.93)	.012	1.44 (1.01–2.06)	.045
Close relationships: 0–1	1		1	
2–3	0.52 (0.30–0.91)	.021	0.51 (0.25–1.03)	.059
4–5	0.44 (0.25–0.77)	.004	0.44 (0.22–0.89)	.023
6–9	0.41 (0.24–0.68)	.001	0.39 (0.20–0.75)	.005
≥10	0.45 (0.26–0.77)	.003	0.44 (0.23–0.85)	.015

*Notes*: ^a^Male is the reference group.

^b^No illness is the reference group.

^c^No mobility impairment is the reference group.

^d^Low depressive symptoms is the reference group.

^e^Married is the reference group.

The fourth sensitivity analysis involved the augmented definition of dementia. Incidence of severely impaired cognitive function was 0.4% at baseline (2004), rising to 1.2% in Wave 6 (2012). The analysis was based on 6,651 instead of 6,677 participants because 26 had scores on the combined cognition measure below threshold at baseline. These individuals were removed from the analysis. There were 340 incident cases using the augmented dementia definition over the follow-up period, giving an age-adjusted incidence over the 6.25-year period of 5.5% overall, and 8.6% in participants aged ≥65 years. In the full model that included all the social relationship variables, there were independent associations between loneliness and dementia incidence (OR 1.24, 95% CI 1.002–1.54, *p* = .048), marriage and dementia incidence (OR 1.80, 95% CI 1.39–2.33), and number of close relationships and dementia (OR 0.56 to 0.40), but not between social isolation and dementia (Table 4). These results corroborate findings from the primary analyses.

**Table 4.** Cox Proportional Hazards Regressions of Dementia Incidence With Enhanced Definition of Dementia (2006–2012) on Social Relationship Variables

	Model 1	Model 2	Model 3	Model 4	Model 5
Sex^a^	1.20 (0.94 to 1.52)	1.21 (0.95–1.53)	1.20 (0.94–1.52)	1.23 (0.97–1.56)	1.22 (0.96–1.55)
Age: 52–59 years	1 [ref]	1 [ref]	1 [ref]	1 [ref]	1 [ref]
60–69	1.33 (0.85 to 2.09)	1.34 (0.65–2.10)	1.35 (0.86–2.12)	1.33 (0.84–2.08)	1.34 (0.86–2.12)
70–79	4.13 (2.75 to 6.22)*	4.18 (2.77–6.29)*	4.21 (2.79–6.33)*	4.07 (2.70–6.12)*	4.14 (2.75–6.24)*
≥80	10.57 (6.85 to 16.29)*	10.74 (6.95–16.59)*	10.82 (7.02–16.68)*	10.51 (6.82–16.20)*	10.79 (6.98–16.67)*
Education: Lower	1 [ref]	1 [ref]	1 [ref]	1 [ref]	1 [ref]
Intermediate	0.74 (0.57–0.94)*	0.74 (0.58–1.95)*	0.75 (0.58–0.96)*	0.75 (0.58–0.96)*	0.76 (0.59–0.98)*
Higher	0.81 (0.57–1.13)	0.82 (0.58–1.15)	0.81 (0.58–1.14)	0.81 (0.57–1.14)	0.82 (0.58–1.15)
Wealth (decile)	0.94 (0.89–0.98)*	0.94 (0.89–0.98)*	0.94 (0.90–0.98)*	0.94 (0.90–0.99)*	0.94 (0.90–0.99)*
Hypertension^b^	1.21 (0.97–1.52)	1.21 (0.97–1.51)	1.22 (0.97–1.52)	1.24 (1.00–1.55)	1.24 (1.00–1.55)
Diabetes^b^	1.23 (0.90–1.69)	1.23 (0.90–1.70)	1.25 (0.91–1.72)	1.19 (0.86–1.63)	1.21 (0.88–1.66)
Stroke^b^	1.31 (0.92–1.89)	1.30 (0.90–1.87)	1.31 (0.91–1.88)	1.28 (0.89–1.85)	1.28 (0.89–1.84)
Coronary heart disease^b^	1.04 (0.78–1.38)	1.03 (0.77–1.37)	1.03 (0.77–1.37)	1.08 (0.81–1.43)	1.06 (0.79–1.40)
Cancer^b^	0.85 (0.56–1.27)	0.84 (0.56–1.27)	0.86 (0.57–1.29)	0.86 (0.57–1.29)	0.87 (0.58–1.31)
Mobility^c^	1.26 (0.96–1.65)	1.27 (0.86–1.69)	1.21 (0.92–1.58)	1.28 (0.98–1.67)	1.23 (0.94–1.62)
Depression^d^	0.95 (0.57–1.59)	0.91 (0.54–1.53)	0.78 (0.45–1.33)	0.93 (0.56–1.56)	0.78 (0.45–1.34)
Cognition	0.32 (0.27–0.37)*	0.32 (0.27–0.37)*	0.32 (0.28–0.38)*	0.32 (0.27–0.37)*	0.32 (0.27–0.38)*
Marital status^e^	1.58 (1.23–2.04)*	1.58 (1.23–2.04)*	1.71 (1.32–2.21)*	1.72 (1.22–2.22)*	1.80 (1.39–2.33)*
Social isolation: 0		1 [ref]			1 [ref]
1		1.08 (0.85–1.37)			1.01 (0.80–1.28)
2		1.08 (0.67–1.72)			1.00 (0.62–1.60)
3		1.66 (0.77–3.57)			1.38 (0.64–2.99)
Loneliness			1.34 (1.08–1.65)*		1.24 (1.00–1.54)*
Close relationships: 0–1				1 [ref]	1 [ref]
2–3				0.56 (0.37–0.85)*	0.56 (0.37–0.85)*
4–5				0.56 (0.37–0.84)*	0.57 (0.38–0.86)*
6–9				0.50 (0.34–0.73)*	0.52 (0.35–0.76)*
≥10				0.37 (0.25–0.56)*	0.40 (0.26–0.61)*

*Notes*: Adjusted hazards ratios with 95% confidence intervals *N* = 6,651.

^a^Male is the reference group.

^b^No illness is the reference group.

^c^No mobility impairment is the reference group.

^d^Low depressive symptoms is the reference group.

^e^Married is the reference group.

*p < .05.

The fifth set of sensitivity analyses related to the computation of the social isolation index, and is summarized in Supplementary Table 4. The association between social isolation and dementia index remained nonsignificant when marital/cohabiting status was added to the index, and when the threshold of frequency of contact with children, other family and friends was either increased or decreased. These analyses suggest that the lack of a relationship between social isolation and dementia was not reliant on a specific threshold for defining isolation.

## Discussion

This analysis investigated risk of dementia in relation to structural and qualitative aspects of middle-aged and older people’s social relationships. In multivariable analyses, loneliness was positively and independently related to increased risk of developing dementia, whereas being married and having more close relationships were each independently associated with a reduced dementia risk. By contrast, social isolation defined as extent of contact with family and friends was not related to development of dementia. These findings were confirmed in logistic regression as well as proportional hazards regression, in analyses restricted to people who did not die during the course of the study, and when we analyzed cases defined by an augmented dementia criterion that incorporated severely impaired cognitive performance in addition to physician diagnoses and informant ratings. The lack of association with social isolation remained when different thresholds of frequency of contact were tested.

Our findings are broadly consistent with two previous studies that have assessed loneliness and social isolation simultaneously. Wilson and colleagues (2007) followed 823 participants in the Rush Memory and Aging Project over a 4-year period, and found that loneliness predicted dementia onset independently of measures of social network size and social participation. Network size was not associated with dementia risk, but reduced social participation was. In the Amsterdam Study of the Elderly (AMSTEL), dementia over a 3-year period was predicted by positive scores on a simple rating of loneliness, whereas social isolation was not (Holwerda et al., 2014).

The findings are apparently at variance with studies indicating that social networks are associated with cognitive decline and dementia incidence independently of covariates (Crooks et al., 2008; Fratiglioni et al., 2000; Saczynski et al., 2006; Stoykova et al., 2011). One explanation may be that some studies have used composite measures of social networks that included marital status and social support. We analyzed marital status separately, confirming that it did predict future dementia. We adjusted for a wider range of covariates than in many studies, including depressive symptoms and mobility impairment. Case ascertainment took place every 2 years, allowing for more precision in timing than in studies that relied on a single follow-up assessment conducted several years after baseline (Fratiglioni et al., 2000; Holwerda et al., 2014). Our findings suggest that structural aspects of social activity such as the frequency of contacts outside the marital relationship are less important than perceptions of closeness.

Our measure of social isolation differed in several respects from those applied in other studies of dementia risk. The focus of the assessment was on isolation rather than high-frequency social contact, so we used the cut-off of less than monthly contact with friends, children and relatives as an indicator of social isolation. This threshold is the same as that used in other well-known measures such as the Social Network Index (Cohen et al., 1997) and the Berkman-Syme social network measure (Berkman & Syme, 1979). We excluded marital status from the index since there is a consistent literature relating marriage to reduced dementia risk (Fratiglioni et al., 2000; Sundstrom, Westerlund, & Kotyrlo, 2016), and we wished to explore other social ties. It is notable in Supplementary Table 1 that social isolation was uncorrelated with marital status, indicating that marriage neither augmented nor reduced the extent of contact with others. Additionally, the sensitivity analyses in which the threshold of frequency of contact for defining isolation was either increased or decreased did not lead to a different result.

Given the observational nature of our study, there is a potential risk of reverse causality. Participants in the early stages of cognitive decline may withdraw from close relationships or be rejected from relationships, leading to an apparent longitudinal association with future dementia. We tried to protect against this possibility by measuring cognition and other risk factors at baseline, and demonstrating that the associations of loneliness and close relationships with future dementia were independent of factors that might influence these states. Additionally, our analysis excluding cases that emerged in the first 4 years after baseline assessments addressed the possibility of incipient dementia affecting social relationships. The observation that associations were maintained after these more immediate cases were excluded adds weight to the temporal sequence. Nevertheless, given that dementia develops over many years, relevant processes may have started before the baseline measures of social relationship variables.

The findings have implications for the relevance of some of the pathways linking loneliness and social isolation with dementia outlined earlier. Associations between marital status, loneliness and number of close relationships were independent of hypertension and diabetes, as well as manifest cardiovascular disease. This suggests that connections in this study were not mediated by cardiovascular risk processes. We also took account of depressive symptoms, since feelings of loneliness are also known to be associated with depression that is itself related to dementia risk (Cacioppo & Hawkley, 2009; Kaup et al., 2016). The relationship of loneliness with dementia risk in our analysis was independent of depression, corroborating earlier findings (Holwerda et al., 2014; Wilson et al., 2007). Other possible mechanisms include psychobiological processes associated with loneliness and close relationships, including inflammatory responses and neuroendocrine dysregulation (Kiecolt-Glaser, Guoin, & Hantsoo, 2010; Cacioppo et al., 2015). Additionally, we did not model health behaviors such as sedentary behavior, smoking, or body weight that are influenced by social relationships and are associated with cognitive decline (Beckett, Ardern, & Rotondi, 2015; Zhong et al., 2015).

An important limitation of these analyses is that dementia was based primarily on doctor diagnoses. Although these were supplemented by informant ratings of cognitive decline on a standardized scale, it is likely that cases were missed. The age-adjusted incidence of dementia was likely an underestimate of the true level, given that a substantial number of dementia cases in England may not be formally diagnosed (Brayne & Davis, 2012). We therefore supplemented physician diagnoses and informant ratings with cognitive performance measures. Although ELSA has included a number of tests including measures of verbal fluency, prospective memory, fluid intelligence and speed and attention (Llewellyn, Lang, Langa, & Huppert, 2008), none of these has been assessed in all waves of data collection. Consequently, we were limited to the two domains of memory and time orientation. Nevertheless, the findings with the augmented definition of dementia were similar to those in the main analyses, strengthening our confidence in the findings.

Several participants did not provide data on dementia during follow-up. They were older, less wealthy, and less cognitively able at baseline compared with the analytic sample. This pattern was not related to loneliness or the social isolation measures at baseline, so we can only speculate about the affect this pattern might have on our results.

There is a possibility of misclassification of cases based on reported physician diagnoses, perhaps because of memory failures. Although we were unable to verify the accuracy of the diagnostic information in this study, self-reports of other conditions, including stroke, have been found to correspond closely with physician diagnoses, even in the presence of overt cognitive impairment (Jin et al., 2010). A misclassification bias is unlikely to account for our results given their consistency with findings from studies that used more objective clinical evaluations (Holwerda et al., 2014; Wilson et al., 2007). Additionally, misclassification would lead to genuine cases being falsely defined as non-cases. This would have the effect of increasing the difficulty of detecting a real association with social relationship variables. We were not able to distinguish Alzheimer’s disease from other forms of dementia. Finally, we selected covariates not only because they were associated with dementia risk, but because they could potentially confound the relationship between social relationships and dementia risk. As an example, limitations in mobility were taken into account because of their affect on social relationships and the frequency of contact. Many other measures could have been considered, including certain health behaviors. However, rather than being true confounders, some of these factors might actually operate as mediators on the causal pathway linking loneliness and social factors to cognitive impairment.

This investigation of older participants in the ELSA demonstrated that several aspects of social relationships in later-life were independently associated with the development of dementia; loneliness predicted greater dementia risk, whereas being married and having many close relationships with friends and family were related to a lower risk of dementia. Further epidemiological research is needed to understand the possible causal nature of these associations, including the likely underlying mechanisms. There has been a growth of interest in intervention studies designed to alleviate loneliness and enhance social engagement, with potentially promising findings (Cohen-Mansfield & Perach, 2015; Dickens, Richards, Greaves, & Campbell, 2011; Masi, Chen, Hawkley, & Cacioppo, 2011). Whether these have a consistent affect on cognitive function is not yet known. It remains to be discovered whether policies and interventions that help improve older people’s sense of belonging or cement close relationships, could effectively delay or prevent the onset of dementia.

## Funding

This work was supported by The Promoting Independence in Dementia (PRIDE) study funded by the UK Economic and Social Research Council (ESRC) and National Institute for Health Research (Grant ES/L001802/1). The English Longitudinal Study of Ageing is funded by the National Institute on Aging (Grant RO1AG7644) and by a consortium of UK government departments coordinated by the ESRC. The data are lodged with the UK Data Archive. A. Steptoe is supported by the British Heart Foundation. The funders of this study had no role in the study design, data collection, data analysis, data interpretation, or writing of the report.

## Conflict of Interest

None declared.

## Supplementary Material

gbx087_suppl_Supplementary-Material
