# Supplementary material for: Loneliness, Social Integration, and Incident Dementia Over 6 Years: Prospective Findings From the English Longitudinal Study of Ageing
Source: J Gerontol B Psychol Sci Soc Sci. 2017 Jun 27;75(1):114–24. doi: 10.1093/geronb/gbx087 (PMC6909434; doi:10.1093/geronb/gbx087)
Supplement: gbx087_suppl_Supplementary-Material [file gbx087_suppl_supplementary-material.docx]

**Supplementary table 1 Intercorrelations between social relationship variables**

|  | Social isolation | Loneliness | N close relationships |
| --- | --- | --- | --- |
| Marital status | 0.01 | -0.31 | 0.15 |
| Social isolation |  | 0.09 | -0.19 |
| Loneliness |  |  | -0.23 |

Note: all correlations except between marital status and social isolation were significant at p<0.001

**Supplementary table 2 Binary logistic regression of the incidence of dementia (2006 – 2012) on social relationship variables**

|  | **Adjusted odds ratio**  **(95% C.I.)** | **p** |
| --- | --- | --- |
| Sex^1^ | 1.34 (0.98 – 1.83) | 0.070 |
| Age: 52-59 years  60-69  70-79  ≥80 | 1  1.71 (0.89 – 3.32)  5.84 (3.18 – 10.73)  13.45 (7.12 – 25.38) | 0.11  <0.001  <0.001 |
| Education: Lower  Intermediate  Higher | 1  0.94 (0.67 – 1.31)  0.99 (0.64 – 1.56) | 0.69  0.99 |
| Wealth (decile) | 1.00 (0.95 – 1.06) | 0.93 |
| Hypertension^2^ | 1.32 (0.98 – 1.78) | 0.067 |
| Diabetes^2^ | 1.41 (0.93 – 2.14) | 0.10 |
| Stroke^2^ | 1.44 (0.91 – 2.14) | 0.12 |
| Coronary heart disease^2^ | 1.03 (0.71 – 1.50) | 0.88 |
| Cancer^2^ | 1.11 (0.68 – 1.81) | 0.68 |
| Mobility^3^ | 1.15 (0.81 – 1.64) | 0.45 |
| Depresssion^4^ | 0.77 (0.37 – 1.58) | 0.47 |
| Cognition | 0.35 (0.27 – 0.44) | <0.001 |
| Marital status^5^ | 1.92 (1.36 – 2.71) | <0.001 |
| Social isolation: 0  1  2  3 | 1  1.00 (0.73 – 1.36)  0.81 (0.43 – 1.53)  1.11 (0.35 – 3.52) | 0.98  0.52  0.86 |
| Loneliness | 1.36 (1.03 – 1.80) | 0.031 |
| Close relationships: 0-1  2-3  4-5  6-9  ≥10 | 1  0.52 (0.30 – 0.91)  0.41 (0.24 – 0.71)  0.39 (0.24 – 0.65)  0.43 (0.25 – 0.73) | 0.020  0.001  <0.001  0.002 |

^1^ Male is the reference group; ^2^ No illness is the reference group; ^3^ No mobility impairment is the reference group;

^4^ Low depressive symptoms is the reference group; ^5^ Married is the reference group.

**Supplementary Table 3 Cox proportional hazards regressions of dementia incidence (2006 – 2012) on social relationship variables, excluding fatalities**

|  | **Adjusted hazards ratio**  **(95% C.I.)** | **p** |
| --- | --- | --- |
| Sex^1^ | 1.19 (0.82 – 1.73) | 0.36 |
| Age: 52-59 years  60-69  70-79  ≥80 | 1  1.59 (0.80 – 3.19)  4.78 (2.51 – 9.10)  14.05 (7.06 – 27.95) | 0.19  <0.001  <0.001 |
| Education: Lower  Intermediate  Higher | 1  0.79 (0.53 – 1.19)  0.71 (0.41 – 1.22) | 0.26  0.21 |
| Wealth (decile) | 0.95 (0.88 – 1.02) | 0.18 |
| Hypertension^2^ | 1.26 (0.89 – 1.79) | 0.19 |
| Diabetes^2^ | 1.44 (0.90 – 2.29) | 0.13 |
| Stroke^2^ | 1.53 (0.90 – 2.61) | 0.11 |
| Coronary heart disease^2^ | 1.09 (0.69 – 1.74) | 0.71 |
| Cancer^2^ | 1.08 (0.59 – 1.96) | 0.80 |
| Mobility^3^ | 1.14 (0.76 – 1.72) | 0.53 |
| Depresssion^4^ | 0.74 (0.32 – 1.70) | 0.47 |
| Cognition | 0.34 (0.26 – 0.45) | <0.001 |
| Marital status^5^ | 2.03 (1.34 – 3.07) | 0.001 |
| Social isolation: 0  1  2  3 | 1  1.20 (0.83 – 1.73)  1.04 (0.51 – 2.08)  1.26 (0.38 – 4.22) | 0.35  0.92  0.70 |
| Loneliness | 1.42 (1.02 – 1.97) | 0.039 |
| Close relationships: 0-1  2-3  4-5  6-9  ≥10 | 1  0.38 (0.21 – 0.70)  0.38 (0.21 – 0.69)  0.30 (0.17 – 0.52)  0.31 (0.18 – 0.56) | 0.002  0.001  <0.001  <0.001 |

^1^ Male is the reference group; ^2^ No illness is the reference group; ^3^ No mobility impairment is the reference group;

^4^ Low depressive symptoms is the reference group; ^5^ Married is the reference group.

**Supplementary table 4 Bivariate associations between different classifications of social isolation and dementia incidence**

|  | **No dementia**  **(n = 6457)** | **Dementia**  **(n = 220)** | **p difference** |
| --- | --- | --- | --- |
| Primary social isolation  index (< 1/month): 0  1  2  3 | 3899 (60.4%)  2057 (31.9%)  423 (6.6%)  78 (1.2%) | 130 (59.1%)  74 (33.6%)  12 (5.5%)  4 (1.8%) | 0.73 |
| Social isolation index including  marital status: 0  1  2  3  4 | 2678 (41.5%)  2598 (40.2%)  985 (15.3%)  167 (2.6%)  29 (0.4%) | 74 (33.6%)  104 (47.3%)  34 (15.5%)  7 (3.2%)  1 (0.5%) | 0.10 |
| Social isolation  index (< 1/week): 0  1  2  3 | 2543 (39.4%)  2397 (37.1%)  1180 (18.3%)  337 (5.2%) | 100 (45.5%)  79 (35.9%)  27 (12.3%)  14 (6.4%) | 0.11 |
| Social isolation index  (< 2/3 months): 0  1  2  3 | 4551 (70.5%)  1723 (26.7%)  164 (2.5%)  19 (0.3%) | 152 (69.1%)  57 (25.9%)  10 (4.5%)  1 (0.5%) | 0.31 |
|  |  |  |  |
